# Psychometric Properties of the Spanish Version of the Goal Orientation Scales in Ecuadorian Undergraduate Students

**DOI:** 10.3389/fpsyg.2020.597934

**Published:** 2020-11-30

**Authors:** Segundo Napoleón Barreno, Alejandro Veas, Leandro Navas, Juan Luis Castejón

**Affiliations:** ^1^Central University of Ecuador, Quito, Ecuador; ^2^Department of Developmental Psychology and Didactics, University of Alicante, Alicante, Spain

**Keywords:** goal orientation scales, exploratory factor analysis, confirmatory factor analysis, factorial invariance, multidimensional Rasch analysis, undergraduate population

## Abstract

The present study aims to analyze the psychometric properties of the Goal orientation Scales (GOS; Skaalvik, 1997) in a sample of 2,170 Ecuadorian undergraduate students (*M* = 21. 97, *SD* = 3.61; 61.6% female). The Exploratory Factor Analysis and Confirmatory Factor Analysis supported the four-factor structure of the GOS, and the scale exhibited an adequate factorial invariance for gender. The multidimensional Rasch analysis revealed that one item showed misfit, and the distribution of items did not correspond well with the levels of achievement goals. The current research addresses a formal gap related to the validation of the GOS in a Latin American country and provides advanced psychometric information to further improve the scale for its application to Spanish-speaking samples.

## Introduction

Achievement goal theory (AGT) has become an important framework that is applied to explain achievement- or competence-related behaviors of an individual (Elliot, 1999; Maehr and Zusho, 2009). In this sense, a goal orientation is an integrated pattern of beliefs, engagements, and actions that underwrite many successful undertakings, including educational endeavors (Ames, 1992). Goal orientations integrate crucial variables that help researchers better understand learning and achievement. They may also help identify students' engagement levels as well as their reasons for low engagement or refusal to perform specific tasks.

One of the most employed approach to understanding goal orientations included mastery vs. performance and approaching vs. avoiding tendencies. When both the direction (approaching vs. avoiding) and orientation (mastery vs. performance) are considered, as can be seen in the 2 × 2 models, four types of goal orientations arise: (1) mastery-approach goals (MApG), (2) mastery-avoidance goals (MAvG), (3) performance-approach goals (PApG), and (4) performance-avoidance goals (PAvG) (Elliot, 1999; Pintrich, 2000; Elliot and McGregor, 2001; Harackiewicz and Linnenbrink, 2005; Bartels et al., 2009). MApG goals apply to students who want to master and learn tasks. PApG goals apply to students who feel superior to others, obtain better qualifications, or stand out in the class. MAvG goals apply to students whose goals are simply to avoid mistakes rather that intrinsically complete any given task well. Finally, students with PAvG goals emphasize not being “less” than others, not obtaining the worst qualifications, or not being the worst student in the class. For example, it has been shown that the approach-avoid motivation system plays an important role in cognitive ability and self-regulation in undergraduate students (Bartels et al., 2009). Similarly, it has also been found that MAvG were more negative than MApG and more positive than PApG (Elliot and McGregor, 2001). Goal orientations have been associated with learning strategies, attributional styles, self-regulatory strategies, and academic achievement (Harackiewicz et al., 2000; Suárez et al., 2001; Martínez-Monteagudo et al., 2018).

### Goal Orientation Measures in the Spanish-Speaking Context

Different instruments to assess students' goals have been employed in Spain. For example, the Study Goal Questionnaire (Núñez et al., 1996) and the Achievement Goal Questionnaire (García et al., 1998), which were both inspired by the Achievement Goal Tendencies Questionnaire (Hayamizu and Weiner, 1991), the Questionnaire for the Evaluation of Academic Goals in Secondary Education (CEMA-II) by Núñez et al. (1997), the MAPE I (Alonso and Sánchez, 1992) and MAPE II (Montero and Alonso, 1992) questionnaires, and the Goal Orientation Scales (GOS; Skaalvik, 1997). The last instrument has the advantage of considering the four types of goals derived from the 2 × 2 models.

Skaalvik (1997) elaborated the GOS in the context of a broader research, with the aim of exploring the two dimensions of ego-goals and how they were related with the approaching and avoiding goal directions. Each scale included between four and seven items, and each item comprised four response categories (true, mostly true, mostly false, and false). The results of the maximum likelihood exploratory factor analysis (EFA) using oblique rotation revealed four factors which corresponded to the expected goal orientation dimensions, with factor loadings higher than 0.5, with one exception. The results indicated the existence of four goal orientations: self-defeating ego, self-enhancing ego, task, and avoidance. Cronbach's alphas were between 0.93 and 0.81.

In the following years, Suárez et al. (2001) translated the scales and implemented a PCA with varimax rotation, supporting the four factors with a 65.4% of the explained variance and Cronbach's alphas between 0.90 and 0.73. Very similar results were obtained by Rodríguez et al. (2001), Rodríguez et al. (2004), and Cabanach et al. (2008). This version of the GOS has been employed in different studies such as those conducted by Valle et al. (2007, 2009, 2015). However, all these studies used Spanish samples who responded to a 5-point Likert scale.

### The Rasch Model in the Context of Goal Orientation Measurement

In recent years, important studies have applied the item response theory (IRT) methods to validate educational measures (Heritage et al., 2020). In fact, Thomas (2011) has claimed that IRT analysis, which focuses on the quality of items in measuring underlying constructs, is a valuable complement to classical test theory approaches (e.g., factor analysis and internal consistency analysis). In this sense, Rasch analysis (Wright and Masters, 1982) will give researchers more confidence in applying the scale in wider contexts. The arithmetical properties of interval scales provide detailed information on the interaction between persons and items (Reckase, 1997).

Indeed, the application of the Rasch model (Rasch, 1960) within goal orientation measures have been extended in recent years, to analyze possible psychometric causes of previous inconsistences between different models of achievement goal theories and other constructs. In this line, Muis et al. (2009) implemented Rasch analyses of the Achievement Goals Questionnaires (AGQ) and the Patterns of Adaptive Learning Scale (PALS) for a sample of undergraduate students, revealing that respondents' ability estimates (level of students agreeing with each item and rating point) varied from “poor” to “good” for both scales. The ACQ was also examined with the Rasch model by Hart et al. (2013). In this case, the sample comprised African American undergraduate students from rural and urban contexts. The results showed acceptable evidence of structural validity; however, there were inconsistencies regarding disordered step categories and low person reliability estimates, mainly due to the targeting of items for these particular samples of students. This implies that the item thresholds do not increase monotonically, and the amount of the latent construct in each item option is not corresponded with the intended order. Moreover, Martin et al. (2008) showed a lack of power discrimination of the mastery goal items included in the Motivational Orientation Scales (MOS) after the implementation of two separate Rasch analyses in School and University students, respectively.

### The Present Study

The present study aimed to validate this instrument and analyze its psychometric properties in Ecuador. Three main objectives were established: (1) to analyze the internal structures of a four-factor model, according to the theoretical model; (2) to see the structural invariance of the model, according to gender; and (3) to analyze the model-data fit of items and participants through a multidimensional Rasch analysis.

## Methods

### Participants

Participants in this study included 2,170 undergraduate students from [BLINDED]: 61.6% female and 38.4% male, aged 17–50 (*M* = 21.97, *SD* = 3.61). Of these students, 55.7% attended classes during the morning hours, and 44.3% during the evenings. The students who reported that they studied part-time and worked part-time comprised 22.6%, whereas 77.4% were full-time students. Simultaneously, students pursued different undergraduate degrees such as Social Sciences (12.17%), Computer (9.63%), Language or Literature (9.68%), English-language (11.20%), Mathematics (9.63%), Kindergarten (14.98%), Plurilingual Pedagogy (7.47%), and Psychology (18.66%). To select the sample, a proportionate stratified random sampling method, with subsamples proportional to the number of students in each Grade, was used.

### Instrument

The Spanish version of the GOS (Skaalvik, 1997), as articulated by Rodríguez et al. (2001), was employed in this study. Our version of the instrument comprised 21 items (one item was removed from the original scale, because it was not significantly explained by any of the factors) and four subscales. The first subscale measured MApG, and it was assessed by items 3, 5, 9, 11, 15, and 18 (e.g., “It is important to learn new things in class,” and “I like learning interesting things in class”). It represented the extent to which students focused on the desire to learn, increase their knowledge, and develop their capacities. The second subscale measured PApG, and it was assessed by items 1, 4, 13, 16, and 19 with statements such as “In this undergraduate degree, I always try to do better than other students” and “It is important to me to know how to do the tasks that other students do not.” This subscale indicated the extent to which students wanted to prove that they were more capable and had achieved more than others. The third subscale measured MAvG and was assessed by items 6, 8, 10, 14, 17, and 20 with statements such as “In this faculty, it is important to me not to seem foolish” and “I am worried about being ridiculed in class.” It measured the extent to which students focused on avoiding appearing incompetent or being negatively judged by others. The fourth subscale measured PAvG and was assessed by items 2, 7, 12, and 21 with statements such as “I prefer subjects where there is no need to work” and “I try to avoid difficult tasks or subjects.” It measured the extent to which students focused on avoiding effort and academic tasks.

Students answered using a modified 5-point Likert scale (the original scale has only four points). In the Spanish versions of Rodríguez et al. (2001) and Suárez et al. (2001) the Likert scale of response was five points. Furthermore, in the work of Skaalvik (1997), in which the Goal Orientation Scale is validated, it is stated verbatim that “The proposed goal orientations were measured by scales varying from four to seven items stating (p. 73).”

The scale measured the degree of their motivation to study or to avoid studying (1 = never and 5 = always). For the four factors described above, the values for Cronbach's alpha were 0.76, 0.68, 0.85, and 0.60, respectively. These results resembled those of previous studies (Rodríguez et al., 2001).

### Procedure

The present study was approved by the Research Ethics Committee of [BLINDED]. A group of experts—including two Professors of Psychology, two Professors of Spanish-language and a Professor of Language Didactics from the Ecuadorian university system—reviewed the validity of the items as they were stated in the study, to ensure that they could be comprehended by Ecuadorian students; no changes were suggested. Informed consent was then requested from the students, who were instructed to answer with sincerity and without discussing with their partners. Participants responded to the questionnaire in their own classrooms during regular class periods. The process took an average of 20 min.

### Data Analysis

The four-factor structure of the GOS was analyzed using EFA and CFA with different samples, as suggested by Yu and Chang (2018).

Since using Likert type items multivariate normality might be questionable, when multivariate normality is violated Goretzko et al. (2019) recommend conduct Principal Component Analysis at first to reduce the dimensionality of the data, and subsequently use CFA. Therefore, an initial Principal Component Analysis was conducted, and a Promax oblique rotation was used given the expected correlations between the factors/components (Skaalvik, 1997). For these analyses, SPSS-23 was used. For the CFA, polychoric correlation matrices were used, which were constructed by raw data. Descriptive measures and item distribution were measured, and multivariate normality was calculated using Mardia's coefficient. Multivariate non-normality was assessed using the robust maximum likelihood estimation (ML) and the Satorra-Bentler (SB) scaled chi-square value (SBχ^2^) (Satorra and Bentler, 2001).

To test measurement invariance across male and female groups, we use the “multigroup confirmatory factory analysis” (CFA; Byrne, 2008). In multigroup CFA we divide the data set into groups (i.e., male and female) to determine model fit for each group separately, and then make multi-group comparisons. This procedure allows examine whether respondents from different groups interpret the same measure in a conceptually similar way (Bialosiewicz et al., 2013). Establishing invariance involves a series of steps.

First, Configural invariance test allows examine whether the overall factor structure stipulated by the GOS fits well for male and female groups in the sample. The configural model serves as the baseline model.

The next step is to test for metric invariance to examine whether the factor loadings are equivalent across the groups. We constrain the factor loadings to be equivalent across male and female groups. A good multi-group model fit indicates metric invariance—if constraining the factor loadings in this way results in a poorer fit, it suggests that the factor loadings are not similar across age groups.

Ascertaining metric invariance allows to made multi-group comparisons of factor variances and covariances, called structural model, invariance of factor variances and covariances was tested. Metric invariance indicates that each item of the scale loads onto the specified latent factor in a similar manner and with similar magnitude across groups. As so, we can assume that differences in factor variances and covariances are not attributable to gender-based differences in the properties of the scales themselves.

These steps are the typical for obtain *weak invariance*, although we can add two additional phases, the test of the scalar invariance (items intercepts) and the invariance of the factor means, which is known as *strong invariance*.

Since the main objective of this research was to establish the construct validity of the GOS in an Ecuadorian sample, the proposed approach to measurement invariance was the analysis of covariance structures, COVS (Byrne et al., 1989; Byrne, 2008), which only considers parameters representing regression coefficients (i.e., the factor loadings), variances, and covariances (i.e., weak invariance).

Multiple-group confirmatory factor analysis was estimated using EQS version 6.1 (Bentler, 2005). As each step of the implementation of measurement invariance implied the application of more constraints, models were nested within each other. Goodness-of-fit measurements were used to assess how well the observations fit the models, including the Root Mean Square Error of Approximation (RMSEA) and the Robust-Comparative Fit Index (R-CFI). Fit was established based on the cutoff criteria suggested by Marsh et al. (2004): a CFI higher than 0.90 (better if higher than 0.95) and a RMSEA lower than 0.08 are indicative of an adequate model fit.

For the two group analyses, we calculated the adjustment for the RMSEA as recommended for multi-sample analyses (Steiger, 1998). RMSEA (<0.05 “good fit” and <0.08 “acceptable fit”) and R-CFI (<0.95 “good fit” and <0.90 “acceptable fit”) were used to assess the fit of the single models. Assessment of the comparison of the nested model was primarily conducted by examining the significant levels associated with Δχ^2^ and ΔSBχ^2^. This scaled difference test was conducted according to Satorra and Bentler's (2001). In addition, since chi-square is sensitive to the sample size and the degrees of freedom, we use the increase in CFI (ΔCFI), and the mean square error of approximation (RMSEA).

Within the IRT approach, the Rasch model is superior to classic measurement approaches for several reasons. First, the model provides both item difficulties independent of the abilities of the sample, and abilities of the sample independent of item difficulties. Second, it allows for item difficulties and person abilities to be matched along the same latent construct. Third, information about model fit, and person and item reliabilities can be obtained. Fourth, the model allows us to check whether Likert responses across items are similarly interpreted and ordered (Martin et al., 2008; Muis et al., 2009). Considering the instrument as a rating scale, the greater the difficulty of the item, the less likely respondents were to agree with it. The lower the difficulty of the item, the more likely respondents were to agree with (Martin et al., 2008). ConQuest version 2.0 software (Wu et al., 2007) was used to conduct the multidimensional Rasch analysis. The GOS was treated as a multidimensional scale containing four unidimensional subscales. The calibration of the four subscales was simultaneously conducted using the Monte Carlo method. Rasch reliability measures, and infit and outfit statistics, were used to check the quality of the scale. These indexes are the mean values of the squared residuals. Therefore, the larger the squared residual, the larger the misfit between data and model. “Infit statistics give more importance to items that are aligned with a person's ability level, whereas computation for outfit statistics is not weighted” (Bond and Fox, 2007, p. 43). Values of Outfit and Infit mean squares can range from 0 to positive infinity. Values below 1 indicate a model fit that is higher than expected, whereas values >1 indicate a poor model fit.

In addition, the category's function of the rating scale was also examined. Linacre (2002) proposed the following essential criteria: (1) each response category must have a frequency count of at least 10, (2) average measures by category must monotonically advance up the rating scale, (3) each response category should have an outlier-sensitive mean square (outfit MNSQ <2), (4) step calibrations (distance between ratings) must monotonically increase, and (5) advance in step difficulties between step calibrations must be at least 1 logits (for a five-category rating scale) and <5 logits.

## Results

First, the descriptive measures, including skewness and kurtosis, are described in Table 1. Items 3 and 18 show outline values. Moreover, Mardia's coefficient indicates multivariate non-normality (109.36).

**Table 1:** Descriptive statistics for the items of the Goal Orientation Scales (GOS).

	**Items**	**Mean**	**Standard deviation**	**Skewness**	**Kurtosis**
1.	I feel successful at school when I do the work better than other students	3.83	0.85	−0.58	0.60
2.	I like school best when there is no hard work	2.40	1.02	0.43	−0.17
3.	At school it is important for me to learn something new	4.71	0.58	−2.64	9.24
4.	At school I try to score higher than other students	4.45	0.76	−0.52	−0.46
5.	At school I am concerned about improving my skills	4.29	0.76	−0.96	0.75
6.	When I answer questions in class I am occupied by how I am perceived by other students	2.83	1.24	0.03	−0.96
7.	At school I hope that we do not get any homework	2.97	1.04	0.17	−0.26
8.	When I am working on the blackboard I am concerned about what my classmates think about me	2.45	1.21	0.39	−0.85
9.	At school it is important for me to learn to solve the problems we are working with	3.92	0.88	−0.75	0.60
10.	At school it is important for me to avoid looking stupid	2.96	1.44	−0.01	−1.35
11.	At school I like to solve problems by working hard	4.17	0.84	0.90	0.67
12.	At school I hope to avoid any hard questions	2.37	3.64	0.41	0.67
13.	At school it is important for me to manage tasks that other students do not manage	3.64	1.04	−0.64	0.02
14.	The worst thing about doing mistakes at school is that other students may notice	2.46	1.15	0.37	−0.66
15.	What I learn in school makes me want to learn more	4.24	0.82	−1.05	1.23
16.	I always try to do better than other students in my class	3.52	1.13	−0.51	−0.43
17.	When I give a wrong answer in class I am most concerned about what my classmates think about me	2.25	1.19	0.63	−0.55
18.	At school I like to learn something interesting	4.60	0.72	−2.26	6.04
19.	I answer questions in class in order to show that I know more than other students	2.38	1.08	0.44	−0.42
20.	At school I am concerned not to make a fool of myself	2.26	1.16	0.64	−0.45
21.	At school I like to do as little as possible	2.11	1.03	0.65	−0.29

*N = 2,170*.

For the correlation matrix, the Kaiser-Meyer-Olkin measure of sampling adequacy was high KMO = 0.87. According to the PCA results considering 500 students chosen at random from the largest sample (Table 2), four components accounted for 51.3% of the variance. Each item was loaded in each of the four components, according to what was theoretically expected, and based on the results obtained with the original scale by Skaalvik (1997). The factor loadings ranged from 0.85 to 0.58 for Component 1 (MApG); from 0.78 to 0.61 for Component 2 (PApG); from 0.74 to 0.58 for Component 3 (MAvG); and from 0.78 to 0.48 for Component 4 (PAvG). Omega reliability coefficients were 0.89, 0.78, 0.77, and 0.74, respectively.

**Table 2:** Results from a principal component analysis of the Goal Orientation Scales.

**GOS item**	**Factor loading**			
	**1**	**2**	**3**	**4**
**Component 1: Mastery-avoidance goals**				
17. When I give a wrong answer in class I am most concerned about what my classmates think about me.	**0.85**	−0.05	0	−0.05
8. When I am working on the blackboard I am concerned about what my classmates think about me.	**0.85**	0.07	−0.1	0.03
20. At school I am concerned not to make a fool of myself.	**0.83**	0.06	−0.06	−0.01
6. When I answer questions in class I am occupied by how I am perceived by other students.	**0.8**	0.09	−0.04	0.04
14. The worst thing about doing mistakes at school is that other students may notice.	**0.63**	−0.12	0.23	−0.04
10. At school it is important for me to avoid looking stupid.	**0.58**	0.06	0.11	−0.01
**Component 2: Mastery-approach goals**				
18. At school I like to learn something interesting.	−0.01	**0.79**	−0.15	0.14
3. At school it is important for me to learn something new.	0.01	**0.68**	−0.03	0.03
15. What I learn in school makes me want to learn more.	0.01	**0.68**	0.01	−0.02
11. At school I like to solve problems by working hard.	0.06	**0.62**	0.1	−0.06
9. At school it is important for me to learn to solve the problems we are working with.	0.06	**0.62**	0.03	−0.13
5. At school I am concerned about improving my skills.	0.03	**0.61**	0.19	0.07
**Component 3: Performance-approach goals**				
4. At school I try to score higher than other students.	0.04	−0.12	**0.74**	−0.08
13. At school it is important for me to manage tasks that other students do not manage.	−0.02	0.21	**0.68**	0.03
19. I answer questions in class in order to show that I know more than other students.	0.05	−0.17	**0.61**	0.16
1. I feel successful at school when I do the work better than other students.	−0.12	0.25	**0.6**	−0.03
16. I always try to do better than other students in my class.	0.04	0.07	**0.59**	−0.03
Component 4: Performance-avoidance goals				
7. At school I hope that we do not get any homework.	−0.05	0.26	−0.09	**0.78**
2. I like school best when there is no hard work.	−0.1	−0.1	0.14	**0.74**
21. At school I like to do as little as possible.	0.13	−0.08	−0.09	**0.54**
12. At school I hope to avoid any hard questions.	0.17	−0.2	0.08	**0.48**

*N = 500. The extraction method was Principal Component Analysis with Promax with Kaiser rotation. Factor loadings above 0.30 are in bold*.

The CFA implemented for the whole sample exhibited an acceptable model fit: CFI = 0.931, R-CFI = 0.955. Table 3 shows the standardized factor loadings and Table 4 the correlations among the four factors, named according to Skaalvik (1997), obtained in the total sample, and according to gender. All factor loading values were medium to high, and statistically significant. The correlations among the factors were also statistically significant, with different values and different signs, as theoretically expected (Table 4). For the CFA results, Omega reliability coefficients were 0.84 for Factor 1 (MApG); 0.73 for Factor 2 (PApG); 0.89 for Factor 3 (MAvG); and 0.65 for Factor 4 (PAvG).

**Table 3:** Results from the confirmatory factor analysis with the standardized factor loadings of the Goal Orientation Scales obtained in the total sample and according to gender.

**GOS item**	**Factor loading**		
	**Total sample**	**Male**	**Female**
**Factor 1: Mastery-avoidance goals**			
17. When I give a wrong answer in class I am most concerned about what my classmates think about me.	0.87	0.86	0.87
8. When I am working on the blackboard I am concerned about what my classmates think about me.	0.83	0.84	0.83
20. At school I am concerned not to make a fool of myself.	0.79	0.77	0.8
6. When I answer questions in class I am occupied by how I am perceived by other students.	0.79	0.8	0.77
14. The worst thing about doing mistakes at school is that other students may notice.	0.66	0.61	0.7
10. At school it is important for me to avoid looking stupid.	0.54	0.56	0.53
**Factor 2: Mastery-approach goals**			
18. At school I like to learn something interesting.	0.72	0.72	0.72
3. At school it is important for me to learn something new.	0.74	0.76	0.73
15. What I learn in school makes me want to learn more.	0.68	0.66	0.69
11. At school I like to solve problems by working hard.	0.68	0.66	0.68
9. At school it is important for me to learn to solve the problems we are working with.	0.64	0.64	0.64
5. At school I am concerned about improving my skills.	0.65	0.66	0.65
**Factor 3: Performance-approach goals**			
4. At school I try to score higher than other students.	0.58	0.57	0.59
13. At school it is important for me to manage tasks that other students do not manage.	0.8	0.81	0.79
19. I answer questions in class in order to show that I know more than other students.	0.36	0.32	0.39
1. I feel successful at school when I do the work better than other students.	0.66	0.71	0.63
16. I always try to do better than other students in my class.	0.51	0.55	0.5
**Factor 4: Performance-avoidance goals**			
7. At school I hope that we do not get any homework.	0.36	0.37	0.35
2. I like school best when there is no hard work.	0.58	0.56	0.59
21. At school I like to do as little as possible.	0.63	0.62	0.63
12. At school I hope to avoid any hard questions.	0.66	0.7	0.63

*Total sample N = 2,170; Male N = 834; Female N = 1,336. All items factor loadings were significant (p < 0.05)*.

**Table 4:** Correlations between factors extracted from the Confirmatory Factor Analysis.

	**Total sample (*****N=*** **2,170)**			
**Variable/Factor**	**1**	**2**	**3**	**4**
1. Mastery-avoidance	1			
2. Mastery-approach	−0.17^**^	1		
3. Performance-approach	0.20^**^	0.53^**^	1	
4. Performance-avoidance	0.54^**^	−0.52	−0.10^**^	1
	**Male** ***(N=*** **834)**			
**Variable/Factor**	**1**	**2**	**3**	**4**
1. Mastery-avoidance	1			
2. Mastery-approach	−0.20^**^	1		
3. Performance-approach	0.16^**^	0.60^**^	1	
4. Performance-avoidance	0.54^**^	−0.54^**^	−0.20^**^	1
	**Female (*****N=*** **1,336)**			
**Variable/Factor**	**1**	**2**	**3**	**4**
1. Mastery-avoidance	1			
2. Mastery-approach	−0.17^**^	1		
3. Performance-approach	0.23^**^	0.47^**^	1	
4. Performance-avoidance	0.57^**^	−0.50^**^	−0.05	1

* *p < 0.05*;

** *p < 0.01*.

Given the appropriateness of the model, separate CFA processes for males and females were also implemented (Table 5), exhibiting good fit indexes in both sample populations.

**Table 5:** Results of the invariance analysis and measurement of the Goal Orientation Scales according to gender.

**Model**	***χ^2^***				**SB*******χ*****^****2****^						**R-CFI**	**ΔR-CFI**	**RMSEA**	**ΔRMSEA**
	**Value**	***df***	**Δ*df***	**Δχ^**2**^**	***p***	**Value**	***df***	**Δ*df***	**ΔSBχ^**2**^**	***p***				
Males (*n* = 834)	1222.99	183		-		626.13	183		-		0.952	-	0.054	
Females (*n* = 1,336)	1709.33	183		-		840.44	183		-		0.953	-	0.052	
Configural	2816.71	366		-		1418.46	366		-		0.955		0.052	-
Metric	2856.69	383	17	39.98	0.01	1437.94	383	17	19.48	0.30	0.954	−0.001	0.051	−0.001
Partial metric^a^	2842.32	382	16	25.61	0.06	-	-	-	-		0.955	0.001	0.050	−0.002
Structural (co)variances^b^	2854.12	392	10	11.80	0.30	1452.87	392	9	14.93	0.09	0.955	0.001	0.050	−0.002

*SBχ^2^ = Satorra-Bentler scaled chi-square; df = degrees of freedom; R-CFI = robust comparative fit index; RMSEA = root mean square error of approximation; ΔSBχ^2^ = change in model fit in relation to the comparison model*.

a *Compare with configural model*.

b *Compare with partial metric model in χ^2^ and compare with metric model in SBχ^2^*.

To test the invariance of measurement across males and females, we followed the sequence of nested models proposed by Byrne (2008), increasing the constraints from one model to the next. Table 5 summarizes the sequence of models testing for invariance across gender, males (1) and females (2).

The *configural model* (Model 1) was the first step in establishing invariance; the estimation of the parameters of the configural model involved testing whether similar four correlated factors existed across male and female samples, without imposing between-group constraints. This test was passed if a similar four-correlated factor model, with simultaneous parameter estimations in both groups, fit the data. As seen in Table 5, the configural model provided a very good fit; the results (R-CFI = 0.955, RMSEA = 0.052) indicated that the dimensional structure was equal across groups. The configural model served as a baseline model against which to compare more restrictive models.

The *full metric invariance model* (Model 2) was established by adding cross-group constraints to the factor loadings coefficients: that is, a model with all factor loadings constrained to be invariant across males and females. If applying these constraints produced a statistically significant fit increase, then not all factor loadings coefficients were invariant across groups. Employing these constraints, a decrease in fit was obtained from the full metric to the configural or baseline model that was statistically significant for χ^2^ (Δχ^2^ = 39.98, Δ*df* = 17, *p* < 0.05), showing that factor loading coefficients were not invariant across males and females, although this difference did not become statistically significant in the statistic SBχ^2^ (ΔSBχ^2^ = 19.48, Δ*df* = 17, *p* > 0.05). Furthermore, examining other fit indices, as ΔCFI and RMSEA, there was no degradation of the model, ΔCFI = −0.001, which not exceeded the value of 0.01 proposed by Cheung and Rensvold (2002). To determine which parameters were non-invariant across males and females, EQS provides a cumulative multivariate Lagrange Multiplier (LM) test for releasing constraints; the probability values associated with the incremental univariate values (Δχ^2^) were <0.05. The LM test showed that all constraints were non-significant, except for item 14, which belonged to the third factor. If the condition of full factor loading coefficients was not satisfied, it was possible to test *the partial factor loading coefficients invariance model* (Model 3, partial metric), relaxing those constraints that had been shown to be non-invariant in the previous step. Consequently, a partial metric invariant model was implemented after releasing the equality constraints on item 14 factor loadings. This partial metric invariant model was not significantly different from the configural model χ^2^ (Δχ^2^ = 25.61, Δ*df* = 16, *p* > 0.05).

The next test involved *a model with equivalence of factor variances and covariances* across males and females (Structural covariances), also called *structural model* (Byrne, 2008). Regarding the χ^2^ value, the fit of this model was not significantly worse than that of the partial metric invariance model (Δχ^2^ = 11.8, Δ*df* = 10, *p* > 0.05). Regarding SBχ^2^ the structural model was compared with the metric model, since there were no differences between the configurational model and the metric model of invariance of the factor loadings, resulting in non-significant differences between male and female in the varainces and covariances between the factors (ΔSBχ^2^ = 14.93, Δ*df* = 9, *p* > 0.05); and R-CFI was similar (ΔCFI = 0.001).

Thus, the factorial structure and metric of the GOS was similar across males and females, except for item 14 factor loadings which were lower in males (0.608) than in females (0.698), when the criterion of change in Δχ^2^/Δ*df* was used, although the factor loadings remained invariant when the criterion of change in ΔSBχ^2^/Δ*df* , and ΔR-CFI were considered.

With respect the Rasch analysis, As shown in Table 6, all criteria are met except (5), as the distance between category measures between steps 2 and 3 (0.88 logits), and between 3 and 4 (0.84 logits) do not reach 1 logit, which imply that these categories are not well-differentiated.

**Table 6:** Rating scale category fit statistics.

**Category**	**Count**	**Percentage**	**Infit MNSQ**	**Outfit MNSQ**	**Step calibration**	**Category measure**
1	6,552	14	1.01	1.08		−2.27
2	7,416	16	0.86	0.94	−0.77	−0.95
3	10,536	23	0.97	1.01	−0.61	−0.07
4	10,968	24	0.85	0.91	0.26	0.91
5	10,096	22	1.16	1.14	1.12	2.46

*Infit MNSQ, information-weighted mean square; Outfit MNSQ, outlier-sensitive mean square*.

The person separation reliability (analog to Cronbach's alpha) for three subscales exhibited low values: 0.62 for MApG, 0.66 for PApG, and 0.58 for PAvG. Only MAvG obtained a positive value of 0.78. These results imply that the instrument may not be sensitive enough to distinguish between high and low latent trait. With respect to the item-separation reliability, values were all 0.99 for all subscales, which indicated that the person sample was large enough to confirm the item difficulty hierarchy (construct validity) of the instrument.

The item difficulty ranged from −0.60 to 1.19 (Table 7). The most difficult item came from MApG (item 18), whereas the least difficult item came from PApG (item 19). The values of Outfit and Infit MNSQ for most items were >0.6 and <1.4, which can be considered an acceptable range (Lee et al., 2014). Only item 10 showed misfit to the Rasch model; it should be removed or revised in future applications.

**Table 7:** Item difficulty measures, standard errors, infit and outfit mean squares, differential item functioning between males and females.

**Item**	**Item difficulty (SE)**	**MNSQ Infit**	**MNSQ Outfit**
1	−0.59 (0.02)	0.80	0.82
2	0.07 (0.02)	0.91	0.92
3	−1.01 (0.03)	1.06	0.83
4	−0.08 (0.02)	1.20	1.19
5	0.15 (0.02)	0.85	0.87
6	−0.40 (0.02)	0.97	0.99
7	−0.59 (0.02)	1.06	1.09
8	0.10 (0.02)	0.92	0.91
9	0.80 (0.02)	0.82	0.85
10	−0.57 (0.02)	1.71	1.67
11	0.39 (0.02)	0.87	0.89
12	0.09 (0.02)	1.01	1.01
13	−0.33 (0.02)	0.93	0.92
14	0.09 (0.02)	1.05	1.10
15	0.26 (0.02)	0.89	0.88
16	−0.18 (0.02)	1.16	1.15
17	0.39 (0.02)	0.88	0.82
18	−0.60* (0.03)	1.20	1.01
19	1.19* (0.02)	1.16	1.16
20	0.38* (0.02)	0.93	0.97
21	0.42* (0.02)	1.04	1.04

*An asterisk next to a parameter estimate indicate that it is constrained*.

**Figure 1:**
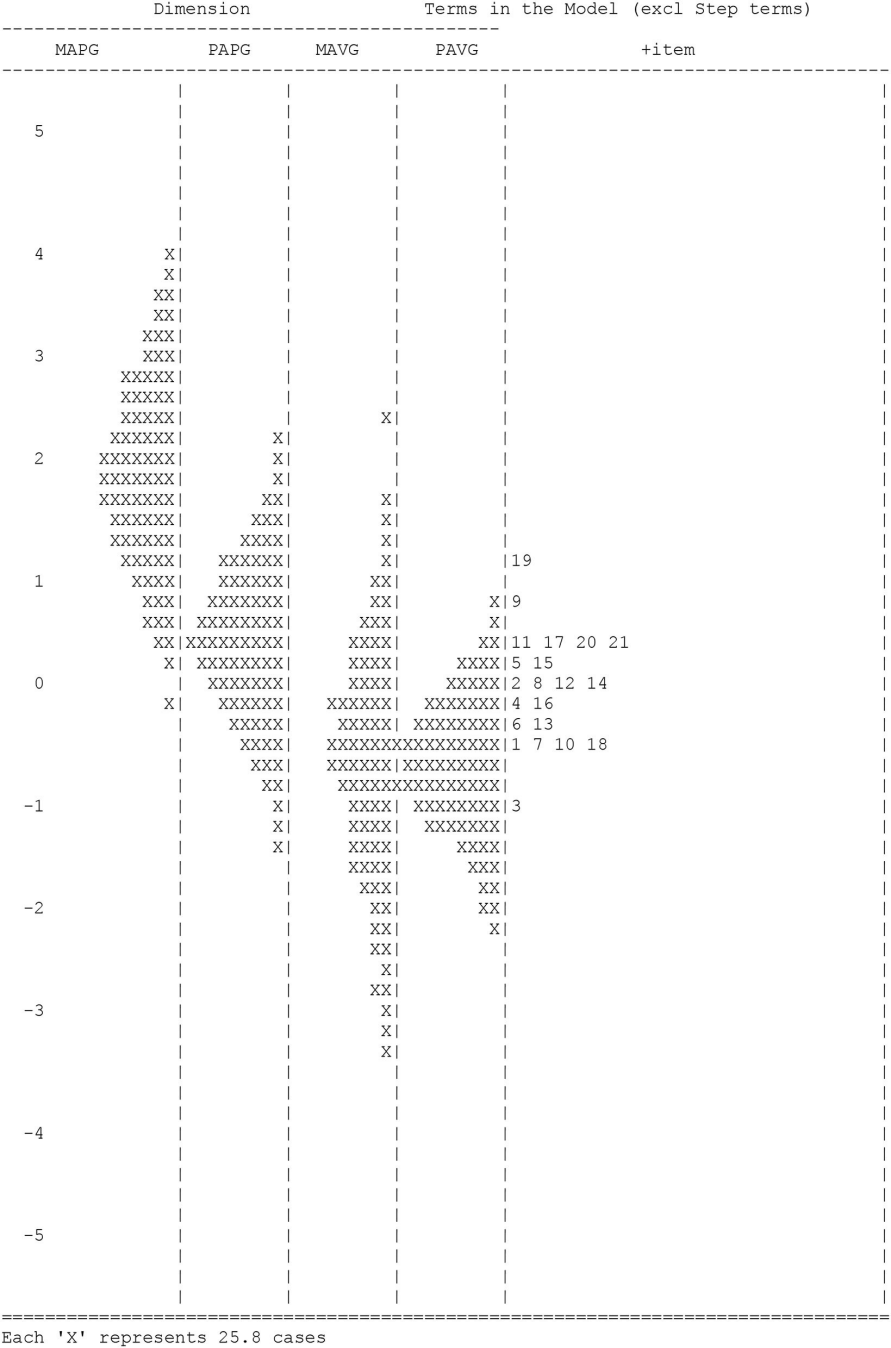
Item-person map.

With the Rasch model, a person's measure can be calibrated from low to high, as item difficulty changes from easy to hard along the same latent trait scale. In the item-person map (see Figure 1), the four continuums on the left side indicate the student's measure in the four dimensions of goal orientations. Students who had higher levels of achievement goals were placed at the top of the continuum and those who had lower levels were placed at the bottom of the continuum. In addition, the items that fell into each of the five dimensions were clustered on the right side. Items with higher difficulty levels were place at the top, and items with lower difficulty levels were placed at the bottom.

The distribution of item difficulties showed that item difficulties matched reasonably well with PApG. However, this distribution was not appropriate for the rest of the dimensions. Person abilities within the MApG dimension were significantly above the item distribution, whereas person abilities in MAvG and PAvG were corresponded well with item difficulties at the top of the continuum. Further, no items matched low-ability persons at the bottom of the continuum. These results are in correspondence with the low person reliability measures described above.

## Discussion

This study aimed to explore the psychometric properties of the Spanish version of the GOS applied to an Ecuadorian undergraduate population. It analyzed the measurement invariance of the model based on gender, and the item-subject interaction within a multidimensional Rasch analysis approach.

First, both EFA and CFA replicated the four original factors described by the author (Skaalvik, 1997). This adequacy of the four-factor structure was consistent with previous studies employing Spanish samples (Rodríguez et al., 2001). Second, the factorial analysis showed that the Spanish version of the GOS had an equivalent factor structure by gender in Ecuadorian undergraduate populations. Although there were some minor inconsistencies related to a non-invariant item factor loading and to the adequacy of the most restrictive model, all inconsistencies were within an acceptable fit range.

Within the multidimensional Rasch procedure, although excellent item reliability measures were reported, three subscales (MApG, PApG, and PAvG) showed poor measurement precision as denoted by inadequate person reliability, which implies that probably more items are necessary to match all the latent trait continuum of the persons.

The majority of the criteria were met for an appropriate response category threshold, and the 5-point rating scale structure found that the response scale categories were used as intended. However, little differentiation was found between response categories 2 and 3, and between 3 and 4. This indicated that the 5-category structure did not function well for the GOS, and a 3-category structure would be strongly recommended by collapsing categories 2, 3, and 4.

In relation to item fit, all of the GOS items showed acceptable fit, except for item 10. Although the item difficulties were within a reasonable range, they were not well-targeted for the sample of Ecuadorian undergraduate students (especially for the MApG, MAvG, and PAvG dimensions), as their ability was not adequately covered by the items. This suggested that more items are required to match students' goal levels at the top and bottom of the continuum. In general terms, similar conclusions were obtained by Hart et al. (2013), as they detected inconsistent step categories with the ACQ items. Moreover, Martin et al. (2008) also detected a lack of discrimination of mastery goals with the MOS, as happens in the present study. It seems that different goal orientation instruments may have similar problems to match item response patterns and item difficulties with persons' levels of goals, specially at the top and the bottom of the latent trait continuum.

The present study has important implications, as it is the first to analyze the psychometric properties of the GOS in a sample of Ecuadorian undergraduate students. From the classical theory perspective, this finding implies the acceptance of the 2 × 2 model as a basic goal orientation model (Elliot and McGregor, 2001; Harackiewicz and Linnenbrink, 2005). Simultaneously, this measure can be crucial for future undergraduate students' employability in terms of work performance (Nerstad et al., 2018). However, from the IRT perspective, a deeper analysis of the items is necessary to improve measurement precision of the instrument with respect Ecuadorian students' range of goal orientations.

Lastly, despite these advantages, it is necessary to address some limitations and lines of inquiry for future research. In concrete terms, the sample of this study was incidental; therefore, a replication of this study is needed using a more reliable sample procedure that assures sample representativeness among the Ecuadorian undergraduate population. Moreover, it is remarkable that the convergent validity of the scale has not been assessed; therefore, future studies should corroborate the construct validity of the GOS, considering the diversity of theoretical models. In this sense, studies should proceed to an extensive measurement of negative motivational values in terms of cost values in specific tasks (Wigfield and Eccles, 1992). The inclusion of both positive and negative value factors may create a more integrated theory framework. Along these lines, Conley (2012) detected seven different motivational profiles for a group of given students, using variables from both the expectancy-value theory and the achievement goal theory, including perception of costs.

## Data Availability Statement

The raw data supporting the conclusions of this article will be made available by the corresponding author under request.

## Ethics Statement

The studies involving human participants were reviewed and approved by Central University of Ecuador. The patients/participants provided their written informed consent to participate in this study.

## Author Contributions

SB: literature review, data collection, and manuscript writing. AV: literature review, statistical analysis, and manuscript writing. LN: literature review, manuscript writing, and manuscript revision. JC: manuscript writing and manuscript revision. All authors: contributed to the article and approved the submitted version.

## Conflict of Interest

The authors declare that the research was conducted in the absence of any commercial or financial relationships that could be construed as a potential conflict of interest.
